# Nutritional deficiency and associated factors among new pulmonary tuberculosis patients of Bale Zone Hospitals, southeast Ethiopia

**DOI:** 10.1186/s13104-019-4786-y

**Published:** 2019-11-19

**Authors:** Bedru Hussien, Mohammedaman Mama Hussen, Abdulwahab Seid, Abduljewad Hussen

**Affiliations:** 1 Department of Public Health, Madda Walabu University, Goba Referral Hospital, Bale-Goba, Ethiopia; 2 Department of Medical Laboratory Science, Madda Walabu University, Goba Referral Hospital, Bale-Goba, Ethiopia

**Keywords:** Nutritional deficiency, Body mass index, Tuberculosis, Bale Zone

## Abstract

**Objective:**

Tuberculosis remains deadliest communicable diseases accountable for health problem among various individuals annually and is related to malnutrition. Addressing nutritional deficiency in Tuberculosis patients is a crucial side of tuberculosis management programme. Therefore, the aim was to assess the magnitude of nutritional deficiency and associated factors among new pulmonary tuberculosis patients of Bale Zone Hospitals, south-east Ethiopia, 2018. Cross-sectional study design was utilized. Data collection was carried out using structured questionnaires and anthropometric measurements. Body mass index was calculated to see nutritional deficiency. Crude and adjusted odds ratios in conjunction with their corresponding 95% confidence intervals were computed. p-value < 0.05 was thought of to declare a result as statistically associated.

**Results:**

Prevalence of nutritional deficiency was 63.2%. The mean Body mass index for all listed participants was 17.86 kg/m^2^. Employment status of the patients, p-value 0.012 (AOR = 1.82; 95% CI 1.14, 2.89) and Khat chewing, p-value 0.02 (AOR = 0.43; 95% CI 0.23, 0.85) were factors independently associated with nutritional deficiency. Prevalence of nutritional deficiency was found to be high. Nutritional support for the impoverished, regular nutritional assessment and dietary counseling are necessary for better treatment outcome and effective Tuberculosis management programme.

## Introduction

Tuberculosis (TB) is a communicable diseases inflicting ill-health among many folks annually and ranks the second leading explanation for death from infectious diseases worldwide, after Human Immune Virus (HIV). It happens in each a part of the planet [1–3]. Deaths from active TB are expected to extend to 5 million a year by 2050 because of increasing populations in countries wherever TB is extremely prevailing, increasing poverty, the unfold of HIV/AIDS, weak health systems and TB program management, insecure funding, and TB drug resistance [4].

Tuberculosis is traditionally related to malnutrition, reduced appetite, low dietary intake, mal-absorption and redoubled caloric demand [5]. However, this relationship is thought to be bidirectional, because the clinical course of the disease results in secondary malnutrition, and malnutrition is additionally a risk issue for the disease [6–8].

Tuberculosis patients have lower body mass index (BMI), muscle mass and subcutaneous stores of fat than others. Tuberculosis patients are 11 times more likely to have a BMI < 18.5 [9]. Undernutrition may worsen the disease, or delay recovery by depressing important immune functions [6, 10, 11]. Severe under-nutrition at diagnosing is related to a twofold higher risk of death. Chronic severe under-nutrition at diagnosing isn’t uncommon, which can persist even after successful treatment in a significant proportion of them [12].

In the study area Bale Zone, TB is one amongst the main health issues of the community [3]. Therefore, under-nutrition during this community is extremely probable for double fold reasons together with agricultural production within the majority of the low lands wherever incidence of TB is comparatively high and therefore the indisputable fact that TB may be a major health problem itself as a result of TB will cause under-nutrition [6].

Tuberculosis treatment alone is commonly not enough to enhance the nutritional status of patients, which underscores the necessity for nutrition screening, assessment, and management as integral parts of TB treatment and care. Therefore, deciding the magnitude of nutritional deficiency among TB patients in certain community as a base for deciding on the necessity of backing poor patients with nutritional support is extremely necessary.

## Main text

### Methods

Facility based cross sectional study design was conducted in Bale Zone which is one of the administrative zones of Oromia Regional State found in south-east Ethiopia from November to April 2018. The zonal town, Robe, is about 430 km far from Addis Ababa, the capital city of Ethiopia. This zone has 21 woredas (3 urban and 18 rural; 12-Agro-pastoralist and 9 Pastoralist weredas). Tuberculosis treatment services are being rendered according to Directly Observed Treatment Schedule, Shorts Course (DOTS) in all the health facilities. The zone had three major ecological zones: highland 14.93%, mid land 21.54%, and low land 63.55%. Agriculture and livestock production is the mainstay of the livelihood of the people [3].

The sample size was determined by using single population proportion formula (${\text{n}}\, = \,{\text{Z}}_{\alpha / 2}^{ 2} {\text{p }}\left( { 1\, - \,{\text{p}}} \right)/{\text{d}}^{ 2}$) by assuming 95% confidence interval, 5% margin of error and taking 37.9% proportion of nutritional deficiency among newly diagnosed TB patients from previous study [13] calculated sample size was 405 patients with non-response rate. The total sample size was proportionally allocated to each hospitals based on the amount of TB clients’ flow of previous year.

Interviewer administered structured questionnaire and anthropometric measurements was used for the data collection. Weights was recorded using regularly calibrated beam balance (+ 100 g precision), with the patient carrying light-weight material. Heights was recorded to the closest centimeter with a studio-meter using standard procedures. Data collection was done using trained nurses with the assistance of investigators.

#### Statistical analysis

Body mass index (BMI in kg/m^2^) was calculated as weight in kilograms divided by the square of height in meters and also the patients was classified into classes supported the BMI cutoffs for weight classes as counseled by the WHO [14]. For children and adolescents <18 years, WHO age and gender-specific BMI z-score tables to classify those with BMI z-score <− 2 as underweight and those with z-score > 2 as overweight [15] were used. SPSS 21 was used for data analysis. Logistic regression was utilized for identifying association between independent and dependent variables. Those variable with a *p*-value less than 0.25 throughout bivariate analysis were taken to the ultimate model for multivariate analysis. Significant association is taken into account with p value less than 0.05.

### Results

#### Characteristics of the study population

A total of 372 new Pulmonary Tuberculosis (PTB) patients were involved in the study which is 91.9% of the intended sample size. Ninety-six (25.8%) of the participants were from Goba Referral Hospital, 70 (18.8%) were from Dello Menna Hospital, 92 (24.7%) were from Robe Hospital, and 114 (30.6%) were from Ginnir Hospital. The mean age of the study participants were 31.55 ± 15.79 years that vary from 2 to 81 years. A bit greater than half of the respondents were male 203 (54.6%) and 164 (44.1%) were from rural areas (Table 1).

**Table 1:** Socio-demographic characteristics of pulmonary tuberculosis patients in hospitals of Bale Zone, Ethiopia, 2018

Variables	Number	Percent (%)	Variables	Number	Percent (%)
Age			Marital status		
< 5	3	0.8	Separated	10	2.7
5–17	42	11.3	Widowed	20	5.4
≥ 18	327	87.9	Ethnicity		
Sex			Oromo	309	83.1
Male	203	54.9	Amhara	53	14.2
Female	169	45.4	Somali	7	1.9
Education			Others	3	0.8
No formal	158	42.5	Religion		
Basic	179	48.1	Muslim	265	71.2
Post Basic	35	9.4	Christian	102	27.4
Employment			Waqeffeta	4	1.1
Working	199	53.5	Others	1	0.3
Not working	172	46.2	Monthly income		
Residence			< 1500 Birr	72	43.4
Urban	165	44.4	1500–3000	55	33.1
Semi urban	43	11.6	> 3000	39	23.5
Rural	164	44.1	Family size		
Marital status			≥ 5	135	36.3
Single	139	37.4	< 5	237	63.7
Married	203	54.6			

#### Nutritional status

**Fig. 1:**
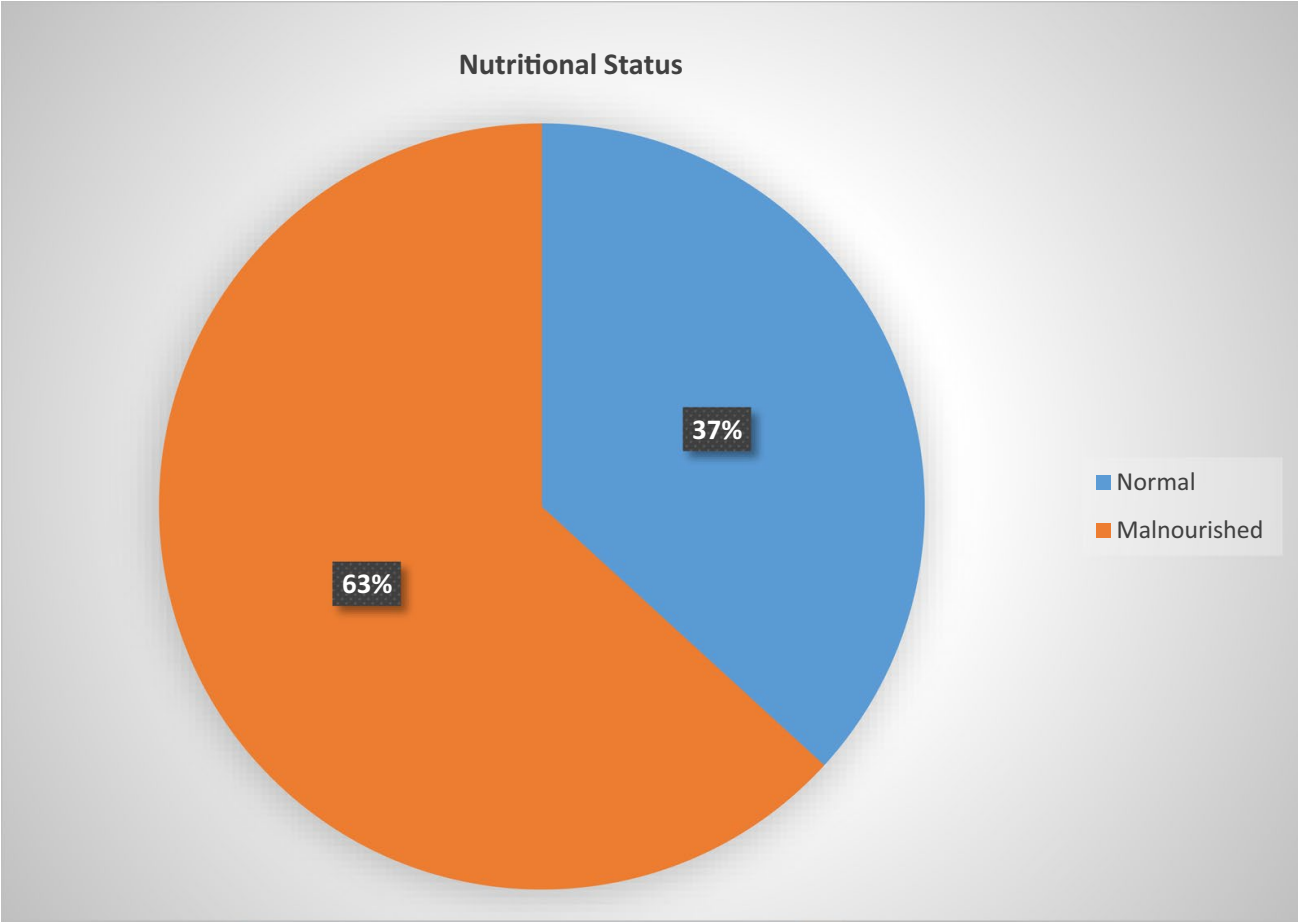
Nutritional status among pulmonary tuberculosis patients attending hospitals in Bale Zone, Ethiopia, 2018

As shown in Fig. 1, the mean BMI for all enrolled participants was 17.86 kg/m^2^; whereas it had been 18.12 kg/m^2^ for males and 17.55 kg/m^2^ for females. Two hundred and thirty-five patients (63.2%) were malnourished at the time of data collection. Among those having nutritional deficiency (ND), 88 (23.7%) have mild ND, 90 (24.2%) severe ND, and 11 (3%) of them were overweight or obese.

#### Health status

Two hundred and forty-four (65.6%) of the patients had smear positive PTB and 337 (90.6%) of them were newly diagnosed. Majority of the study subjects (215, 57.8%) had a length of symptoms before PTB diagnosis was made greater than 1 month. As far as HIV serum status is concerned 43 (11.6%) of the study participants were positive and about 37 (10%) of the subjects have reported to have alternative chronic sicknesses like kidney diseases, asthma, and cardiovascular diseases in conjunction r with tuberculosis.

#### Associated factors

In the bi-variate analysis different variables were found to be considerably related to nutritional deficiency that includes; age, *p*-value 0.13 (COR 95% CI 0.83,3.59); no formal education, *p*-value 0.004 (COR 1.97, 95% CI 1.25–3.12); not working, *p*-value 0.004 (COR 0.53, 95% CI 0.34–0.81); semi urban residence, *p*-value 0.005 (COR 0.52, 95% CI 0.33–0.82); family size greater than or equal to 5, *p*-value 0.009 (COR 1.83, 95% CI 1.16–2.89), and Chat chewing, *p*-value 0.049 (COR 1.91, 95% CI 1.002–3.65), while, ND has no significant association with sex, marital status, HIV serum status, co-morbidity, and length of symptoms before diagnosing was created among others (Additional file 1: Table S1). After controlling the confounding variables, patients with employment status (not working) were 1.73 time more likely to develop nutritional deficiency as compared to those who were working, *p*-value 0.03 (AOR = 1.73, 95% CI 1.07–2.81) and subjects not chewing Khat were less probably to own ND (Table 2).

**Table 2:** Multivariate logistic regression analysis on factors associated with nutritional deficiency among pulmonary tuberculosis patients attending hospitals of Bale Zone, Ethiopia, 2018

Variable	Nutritional deficiency		AOR (95% CI)	*p*-value
	Yes (%)	No (%)		
Age				
≥ 18	202 (54.3)	125 (33.6)	1	
5–17	31 (8.3)	11 (2.96)	1.31 (0.11, 15.27)	0.83
< 5	2 (0.54)	1 (0.27)	0.73 (0.33, 1.60)	0.43
Education				
Basic	115 (30.9)	43 (11.56)	1	
No Formal	103 (27.69)	76 (20.43)	0.45 (0.2, 1.01)	0.054
Post basic	17 (4.57)	18 (4.84)	0.75 (0.035, 1.60)	0.45
Employment				
Working	112 (30.19)	87 (23.45)	1	
Not working	122 (32.88)	50 (13.48)	1.73 (1.07, 2.81)	*0.03*
Residence				
Urban	116 (31.18)	48 (12.9)	1	
Semi urban	27 (7.23)	16 (4.3)	1.17 (0.69, 2.0)	0.57
Rural	92 (24.73)	73 (19.62)	1.11 (0.53, 2.32)	0.80
Family size				
< 5	138 (37.1)	99 (26.61)	1	
≥ 5	97 (26.08)	38 (10.22)	0.64 (0.40, 1.05)	0.08
Khat chewing				
Yes	42 (11.29)	14 (3.76)	1	
No	193 (51.88)	123 (33.06)	0.43 (0.22, 0.84)	*0.01*

Italic values indicate statistical significance of p-value ≤ 0.05

### Discussion

This study confirmed that more than half of PTB patients were suffering nutritional deficiency at the time of beginning treatment. Moreover, employment status of the patients and chewing Khat were factors considerable related to nutritional deficiency among new PTB patients.

The prevalence of nutritional deficiency among PTB patients was 63.2% that is far on top of the study done in Addis Ababa, Ethiopia, 39.7% [13]. This is often as a result of the patients of Addis Ababa were not naïve to treatment, certain proportion of them might have already started recovery as a results of therapy (anti TB treatment) and also the dissimilarity of life style between the two settings.

The current ND finding is comparable with study done in Gondar, Ethiopia 65.4% [13]; whereas less than study done in Sidama, Ethiopia 77.9% [16] that might be as a results of the length of the study that is eleven years ago which will contribute to vary in economic development through time. The opposite studies done in African countries conjointly show similar results; Ghana, 51% [6]; Malawi, 59% [17] and Uganda, 62% [18]. The study done in Gulbarga, India, 62.2% [19]; kuala Lumpur, Malesia, 52.4% [20]; and Bahia, Salvador, Brazil, 50% [21] are also not far from the present finding.

In the study done in central India, moderate to severe under nutrition was 80% for females and 67% for males [12]. The 80% under nutrition for females in India is much higher than that of this study and a lot of astonishingly the degree of under nutrition during this proportion is moderate to severe. This might result to characteristic of the study population that could be a marginalized grouping, with higher rates of impoverishment, illiteracy, and infant and maternal mortality and under-nutrition, than the common Indian population.

Regarding those associated factors assessed for nutritional deficiency, age may contribute to deficiency because of the body demand at completely different stage of life for growth and development. Once any infection is encountered and nutritional intake isn’t acceptable ND is inevitable [5]. During this study the age isn’t commonly distributed across the 3 groups; we’ve got only a few under-fives and adolescents and it’s tough to draw conclusion regarding its association with ND.

Educational status, has a role in ones nutritional status as a result of information of nutrient food stuff, perspective towards food items associated with a lesser probability of avoiding certain food items and good practices of food preparation are all important in having normal range of nutritional standard. The lead to this study concerning education is per this concept; family size, matters in adequacy of food for a house hold member particularly in households with deficiency of food; residence, between rural and urban category may play a role in nutritional status in all probability supported life style including feeding practices.

Employment is a means for generating income for an individual or house hold (HH) to purchase food items also to help in health care. Consistently, this study has shown that employment status, not working, is considerably related to having ND, *p*-value 0.012 AOR 1.82 (95% CI 1.14, 2.89) i.e. people who are not working were 1.82 time more likely to have ND.

Khat chewing may influence nutritional status in several ways among which decrease in appetite and drawing financial deposit and making shortage of money to buy food items are worth mentioning. The current study also shows association between Khat chewing and ND *p*-value 0.02 AOR 0.43 (95% CI 0.23, 0.85) in the multivariable regression which means people who were not chewing Khat were less likely to develop ND or not chewing Khat was protective enough not to develop ND.

With respect to associated factors a study conducted in Ghana [9] also shows that income, educational status and family size were factors associated with under nutrition which is supported by this study. The study done in Addis Ababa [13] also shows functional status of the patient and dietary counseling are associated with ND which is a different aspect of investigating under nutrition. A study in Gondar shows that the prevalence of under nutrition in adult TB patients co-infected with HIV was 71.6% and it is associated with ND [22], but in the current study co-infection was only 11.6% among which 7.53% were having ND and there is no association. This could be due to the fact that the study in Gondar has included high number of TB/HIV co-infected patients who were more exposed to under nutrition because of double burden.

The magnitude of ND in the study population is high where employment status and chat chewing, might have contributed to the problem. Therefore, regular nutritional counseling with focusing on the effect of Khat chewing by frontline, nutritional supplementation to those who are disadvantaged at least for the period of intensive phase for earlier recovery and good adherence to treatment, program set for TB/HIV co-infected cases where support is being rendered can be considered.

## Limitation

The proportion of individuals who respond on monthly financial gain was terribly restricted to the extent that it’s tough to draw conclusion on the impact of nutritional status.

## Supplementary information

**Additional file 1: Table S1.** Bivariate analysis on factors associated with nutritional deficiency among Pulmonary Tuberculosis patients attending hospitals of Bale zone, Ethiopia, 2018.

## Data Availability

All data generated or analyzed during this study are included in this published article [and its additional file].

## Abbreviations

AOR: adjusted odd ratio
BMI: body mass index
COR: crude odd ratio
DOTS: Directly Observed Treatment Schedule, Shorts Course
HC: health centre
HF: health post
HIV: human immune virus
HP: health post
ND: nutritional deficiency
PTB: pulmonary tuberculosis
TB: tuberculosis
