# Supplementary material for: Nutritional deficiency and associated factors among new pulmonary tuberculosis patients of Bale Zone Hospitals, southeast Ethiopia
Source: BMC Res Notes. 2019 Nov 19;12:751. doi: 10.1186/s13104-019-4786-y (PMC6862861; doi:10.1186/s13104-019-4786-y)
Supplement: Supplementary file 1 — Additional file 1: Table S1. Bivariate analysis on factors associated with nutritional deficiency among Pulmonary Tuberculosis patients attending hospitals of Bale zone, Ethiopia, 2018. [file 13104_2019_4786_MOESM1_ESM.docx]

**Table S1: Bivariate analysis on factors associated with nutritional deficiency among Pulmonary Tuberculosis patients attending hospitals of Bale zone, Ethiopia, 2018.**

| **Variable** | **Nutritional Deficiency** | | **COR (95% CI)** | ***P-value*** |
| --- | --- | --- | --- | --- |
|  | **Yes (%)** | **No (%)** |  |  |
| **Age** |  |  |  |  |
| > 18 | 202(54.3) | 125(33.6) | 1 |  |
| 5-17 | 31(8.3) | 11(2.96) | 1.74(0.85,3.59) | 0.13* |
| <5 | 2 (0.54) | 1(0.27) | 1.24(0.11,13.79) | 0.86 |
| **Sex** |  |  |  |  |
| Male | 125(33.6) | 78(20.97) | 1 |  |
| Female | 110(29.57) | 59 (15.86) | 0.86 (0.56,1.31) | 0.48 |
| **Education** |  |  |  |  |
| Basic | 103(27.69) | 76(20.43) | 1 |  |
| No Formal | 115(30.9) | 43(11.56) | 1.97(1.25,3.12) | 0.004* |
| Post basic | 17(4.57) | 18(4.84) | 0.7(0.34,144) | 0.33 |
| **Employmen**t |  |  |  |  |
| Working | 112(30.19) | 87(23.45) | 1 |  |
| Not working | 122(32.88) | 50(13.48) | 0.53(0.34,0.81) | 0.004* |
| **Residence** |  |  |  |  |
| Rural | 116(31.18) | 48(12.9) | 1 |  |
| Semi urban | 27(7.23) | 16(4.3) | 0.7(0.35,1.41) | 0.32 |
| Urban | 92(24.73) | 73(19.62) | 0.52(0.33,0.82) | 0.005* |
| **Marital Status** |  |  |  |  |
| Married | 131(35.22) | 72(19.35) | 1 |  |
| Single | 82(22.04) | 57(15.32) | 0.79(0.5,1.2) | 0.3 |
| Separated | 8(2.15) | 2(0.54) | 2.2(0.4,10.6) | 0.32 |
| Widowed | 14(3.76) | 6(1.61) | 1.28(0.47) | 0.63 |
| **Family size** |  |  |  |  |
| <5 | 138(37.1) | 99(26.61) | 1 |  |
| > 5 | 97 (26.08) | 38(10.22) | 1.83(1.16,2.89) | 0.009* |
| **Type of Pulmonary Tuberculosis** | | |  |  |
| Smear positive | 159(42.7) | 85(22.85) | 1 |  |
| Smear negative | 76(20.43) | 52(13.98) | 1.28(0.82,1.99) | 0.27 |
| **Treatment History** | | |  |  |
| New | 211(56.7) | 126(33.87) | 1 |  |
| Retreated | 24(6.45) | 11(2.96) | 0.77(0.36,1.62) | 0.49 |
| **Co-morbidity** |  |  |  |  |
| No | 26(6.99) | 11(2.96) | 1 |  |
| Yes | 209(56.18) | 126 (33.87) | 0.7(0.34,1.47) | 0.35 |
| **HIV serum** |  |  |  |  |
| Negative | 207(55.65) | 122(40.86) | 1 |  |
| Positive | 28(7.53) | 15(4.03) | 1.1(0.57,2.14) | 0.78 |
| **Alcohol** |  |  |  |  |
| No | 226(60.75) | 128(34.41) | 1 |  |
| Yes | 9(2.42) | 9(2.42) | 0.57(0.22,1.46) | 0.25* |
| **Tobacco** |  |  |  |  |
| No | 214(57.53) | 128(34.41) | 1 |  |
| Yes | 21(5.65) | 9(2.42) | 1.40(0.62,3.14) | 0.42 |
| **Khat** |  |  |  |  |
| No | 193(51.88) | 123(33.06) | 1 |  |
| Yes | 42(11.29) | 14(3.76) | 1.91(1.0,3.65) | 0.049* |
